# Tilapia lake virus: A structured phylogenetic approach

**DOI:** 10.3389/fgene.2023.1069300

**Published:** 2023-04-18

**Authors:** Miriam Abbadi, Andrea Basso, Lorena Biasini, Rosita Quartesan, Alessandra Buratin, Nadav Davidovich, Anna Toffan

**Affiliations:** ^1^ National Reference Laboratory for fish diseases, Istituto Zooprofilattico Sperimentale Delle Venezie, Legnaro, Padova, Italy; ^2^ Israeli Veterinary Services, Bet Dagan, Israel

**Keywords:** TiLV, complete genome, reassortment, phylogenetic signal, Israel

## Abstract

Tilapia Lake Virus (TiLV), also known as *Tilapia tilapinevirus*, is an emerging pathogen affecting both wild and farmed tilapia (*Oreochromis* spp.), which is considered one of the most important fish species for human consumption. Since its first report in Israel in 2014, Tilapia Lake Virus has spread globally causing mortality rates up to 90%. Despite the huge socio-economic impact of this viral species, to date the scarce availability of Tilapia Lake Virus complete genomes is severely affecting the knowledge on the origin, evolution and epidemiology of this virus. Herein, along with the identification, isolation and complete genome sequencing of two Israeli Tilapia Lake Virus deriving from outbreaks occurred in tilapia farms in Israel in 2018, we performed a bioinformatics multifactorial approach aiming to characterize each genetic segment before carrying out phylogenetic analysis. Results highlighted the suitability of using the concatenated ORFs 1, 3, and 5 in order to obtain the most reliable, fixed and fully supported tree topology. Finally, we also attempted to investigate the presence of potential reassortment events in all the studied isolates. As a result, we report a reassortment event detected in segment 3 of isolate TiLV/Israel/939-9/2018 involved in the present study, and confirmed almost all the other events previously reported.

## 1 Introduction

Tilapia Lake Virus (TiLV), also known as *Tilapia tilapinevirus*, is an emerging viral pathogen that in recent years has greatly affected both wild and farmed tilapia inducing important socio-economic effects mostly in developing countries. After carp, tilapia (*Oreochromis* spp.) is the second most farmed fish species for human consumption as it is able to meet the growing demand of protein sources (Ng and Romano, 2013) with a global production of 4.6 million tonnes in 2019 (FAO, 2021). Furthermore, the considerable interest of aquaculture industry towards this species is due to peculiar rearing characteristics, such as a rapid growth rate and annual production, tolerance to high-density aquaculture conditions, resistance to diseases, high nutritional values and great affordability of the final product.

TiLV was firstly discovered in Israel in 2014 (Eyngor et al., 2014; Bacharach et al., 2016a) and since then outbreaks of the disease have been reported in 17 countries across the world, including Ecuador (Ferguson et al., 2014), Colombia (Tsofack et al., 2017), Peru (Pulido et al., 2019), Egypt (Fathi et al., 2017), India (Behera et al., 2018), Thailand (Surachetpong et al., 2017), Malaysia (Amal et al., 2018), and the United States (Ahasan et al., 2020). TiLV infected fish can experience high mortality rates, up to 90%, and exhibit clinical signs associated with the infection such as lethargy, anorexia, exophthalmia, discoloration and abdominal distension, scale protrusion and abnormal swimming behaviour (Tattiyapong et al., 2017; Jansen et al., 2019). In addition, typical internal lesions induced by the disease comprise hepatitis and encephalitis (Fathi et al., 2017).

The aetiological agent of the disease (TiLVD) is described as an enveloped, negative-sense, single-stranded RNA virus with a 10-segment genome ranging from 1,641 to 456 nucleotides (total genome size 10,323 nucleotides) (Eyngor et al., 2014; Bacharach et al., 2016a). To date, among TiLV genome no nucleotide similarity to other sequences present in the public databases have been identified, nor homologies with any known protein.

Initially, the virus was taxonomically assigned to the *Orthomyxoviridae* family due to the viral similarity in terms of morphology and genome organization to the other orthomyxoviruses (Eyngor et al., 2014; del-Pozo et al., 2017). However, the International Committee on Taxonomy of Viruses (ICTV) has recently accepted the proposal of placing the virus as a new species, the *T. tilapinevirus*, under the genus *Tilapinevirus* and family *Amnoonviridae* (Bacharach et al., 2019).

Considering that TiLV has been recognized as a significant infectious agent threatening the development of global tilapia industry, improving the knowledge of how the virus evolves and spreads across the countries is of utmost importance. In the last years, few attempts to study and characterise the TiLV proteome have been made, which enabled the detection of residues from 9 predicted proteins (segment 2–10) by mass spectrometry experiments (Bacharach et al., 2016a). Besides, among all the segments, only the gene on segment 1 was found predicting a protein with a low identity with the RNA-dependent RNA polymerase (RdRP) subunit of influenza C virus (Bacharach et al., 2016a; Bacharach et al., 2016b). Furthermore, studies aiming to identify peptide candidates for vaccine production have recently been carried out reporting promising epitopes in segment 5 and 6 (Lueangyangyuen et al., 2022) and combining residues from segment 9 and 10 (Chamtim et al., 2022). Despite the economic and cultural interest on this fish species, only few TiLV segments (3 and 4) have been evaluated for immunogenic activity using bioinformatics and/or *in vitro* approaches (Piewbang et al., 2021; Abu Rass et al., 2022). Concurrently, several studies attempted to apply phylogenetic analysis, on partial or complete TiLV genomes, in order to trace viral movements in the involved regions (Surachetpong et al., 2017; Pulido et al., 2019; Ahasan et al., 2020; Chaput et al., 2020; Verma et al., 2022). However, overcoming the lack of whole genomes availability is crucial to obtain more accurate information on the history of the virus. Moreover, recent studies also demonstrated that reassortment phenomenon is common for this viral species due to its segmented genetic structure (Chaput et al., 2020; Thawornwattana et al., 2021; Verma et al., 2022). Hence, phylogenetic analysis based on individual genomic segments might fail to describe TiLV evolutionary dynamics, and on the other hand, analysing complete genomes without considering the reassortment events might mislead the interpretation of its history (Chaput et al., 2020; Thawornwattana et al., 2021; Tran et al., 2022; Verma et al., 2022).

In a previous study, tilapia mortality events on 14 Israeli fish farms were investigated during 2017–2018; of the 89 samples analysed, 42% were TiLV positive (Skornik et al., 2020). In this spatiotemporal study, collected samples were diagnosed for the presence of TiLV by SYBR Green-based real-time PCR; reverse transcription PCR (RT-PCR) was performed to amplify and sequence segment 3 of positive samples with no viral isolation. In the present study, we further investigated two tilapia farms from the aforementioned study, which experienced fingerlings mortalities in summer 2018. Firstly, we aimed to confirm whether TiLV was the causative agent of the sudden mortalities. Once confirmed, we carried out complete genome sequencing of the positive isolates and attempted, through a bioinformatic approach, to identify potential genome reassortment events and to suggest a new and useful approach in order to perform reliable phylogenetic analysis of this viral species.

## 2 Materials and methods

### 2.1 Description of disease outbreaks and sample collection

Tilapia fingerlings (*Oreochromis aureus* × *Oreochromis niloticus*) were collected in mid-summer 2018 from two farms, namely, farm J and D (as named by Skornik et al., 2020), located in the north and south of Israel’s “Valley of the Springs.” Farms under investigation had already been reported in a previous study describing several mortality events, which were attributed to TiLV infection (Skornik et al., 2020). While farm J reached a mortality rate of 68% (water temperature 28.8°C), mortality rates in farm D ranged from 50% up to 90% (water temperature 29°C–30°C). Fish samples (brains and livers) were collected at the onset of clinical signs or during mortality events. Samples from 4 fish (farm J) and 8 fish (farm D) were kept at −80°C until further investigations. Frozen organs were sent to the Istituto Zooprofilattico Sperimentale delle Venezie in 2019 for analyses. In particular, target organs (brain and liver) of sampled fish were subject to molecular investigations and viral isolation for TiLV diagnosis. Therefore, no ethical approval was required for this specific study as samples were collected during a natural outbreak of disease.

### 2.2 RNA extraction and TiLV detection by rRT-PCR

Total RNA purification was performed, from brain (*n* = 6), liver (*n* = 5) and pooled brain/liver (*n* = 1) samples, using RNeasy Mini kit (Qiagen, Hilden, Germany) according to the manufacturer’s “Animal Tissues” purification protocol. In order to preserve RNA long-term integrity, 40 units of RNasin^®^ Plus RNase Inhibitor (PromegaWoods Hollow, Road Madison, WI, United States) were added to each sample. All RNA samples were quantified using the Qubit™ RNA BR Assay Kit with the Qubit™ 4 Fluorometer (ThermoFisher Scientific, Waltham, MA, United States) and then normalized at the same concentration in molecular-grade water.

Samples were then subjected to SYBR Green-based reverse transcription real-time PCR (rRT-PCR), targeting segment 3, for TiLV detection (Tattiyapong et al., 2018). In order to gain better chances in performing complete genome sequencing of strains under study, only samples with the highest concentration of viral genetic material underwent viral isolation prior performing Sanger sequencing.

### 2.3 Virus isolation

Frozen samples (liver and brain) from diseased tilapia (*n* = 3) were used for cell culture inoculation. Briefly, organs were homogenized 1:3 ratio with sterile sand in a potter, diluted 1:5 with L-15 Medium (Merck KGaA, Darmstadt, Germany) and clarified by centrifugation for 10 min at 2,800 g. The harvested supernatants were incubated overnight at 4°C with 1% antibiotic and antimycotic solution (10,000 IU/ml penicillin G, 10 mg/ml streptomycin sulphate, 25 μg/ml amphotericin B and 1% of 50 mg/ml solution of Polymyxin B sulphate) (Merck KGaA, Darmstadt, Germany) and inoculated on a 24 h-old E-11 cell monolayer (Iwamoto et al., 2000). The cells, incubated at 25°C, were inspected daily for the appearance of cytopathic effect (CPE) under inverted microscope (Leica DMi1) equipped with camera (FLEXACAM C1, Leica).

### 2.4 Amplification of TiLV genomic segments and sequencing

Isolated TiLV strains, TiLV/Israel/939-9/2018 and TiLV/Israel/939-16/2018, underwent complete genome sequencing by the Sanger sequence-based method. The choice of applying Sanger sequencing, instead of “next-generation” sequencing (NGS) techniques, was based on the fact that this method granted highly accurate, effective and reliable sequencing of small target regions in a simple, rapid and cost-effective way. Indeed, this alternative met well our purposes as we aimed to sequence only two highly positive TiLV isolates with a segmented genome (ranging from 1,641 to 456 nucleotides). These characteristics eased the design of segment-specific primer sets in order to generate relatively small fragments (823 bp or less) and if needed overlapping and covering both strands for sake of completeness. Hence, total viral RNA was isolated from 200 µL of cell-culture supernatant using RNeasy Mini kit (Qiagen, Hilden, Germany) following the manufacturer’s instructions. Viral RNA of each strain was used as a template in 20 different RT-PCR reactions in order to amplify all 10 segments. Segment-specific primer set design was based on a selection of TiLV complete genome sequences, deriving from different countries and available in public databases (Table 1). For each segment, using Geneious software version 2020.1.2 (Biomatters, Auckland, New Zealand) we firstly aligned retrieved sequences with default settings. Then, applying Primer3 tool available in Geneious software, primer sets were designed basing on conserved regions, and whenever possible on untranslated regions (UTRs), in order to cover the full length of the segment (Table 2). All RT-PCR were performed using the OneStep RT-PCR kit (Qiagen, Hilden, Germany) in a final reaction volume of 25 μL containing 5 μl of template RNA, 1X Buffer, 10U of RNasin^®^ Plus RNase Inhibitor (Promega Corporation, Fitchburg, WI, United States), 400 μM dNTPs, 1 μl Enzyme Mix and 0.6 μM specific forward and reverse primer. Cycling conditions consisted of a reverse transcription (RT) step at 50°C for 30 min, RT inactivation and HotStarTaq DNA polymerase activation at 95°C for 15 min; 40 cycles of 1 min denaturation at 94°C, 1 min annealing at the required temperature based on the utilized primer set (ranging from 55°C to 60°C) and 1 min elongation at 72°C; the reaction was terminated with 10 min elongation at 72°C. PCR products were checked for size and purity on 1% agarose gel electrophoresis and then purified with ExoSAP-IT Express (Applied Biosystems by ThermoFisher Scientific, Baltics, UAB, Lithuania) prior Sanger sequencing. For each amplicon, sequencing was performed in both directions using the BrilliantDye™ Terminator (v3.1) Cycle Sequencing kit (NimaGen, Nijmegen, Netherlands). Sequencing reactions products were then cleaned up using the BigDye XTerminator™ Purification Kit (Applied Biosystems by Thermo Fisher Scientific, Bedford, MA, United States) and analyzed on a 16-capillary ABI PRISM 3130xl Genetic Analyzer (Applied Biosystems, Foster City, CA, United States). Sequencing data were assembled and edited using SeqScape™ software version 3.0 (Applied Biosystems).

**TABLE 1:** Reference TiLV complete genomes (ORFs 1 - 10) available at NCBI database. In bold, the genomes sequenced for the current study.

Strain	Acc. Number (ORF 1 - ORF 10)	Country	References
BD-2017^a^	MN939372 - MN939381	Bangladesh	Chaput et al. (2020)
BD-2017-181	MT466437 - MT466446	Bangladesh	Debnath et al. (2020)
BD-2019-E1^a^	MT466447 - MT466456	Bangladesh	Debnath et al. (2020)
BD-2019-E3	MT466457 - MT466466	Bangladesh	Debnath et al. (2020)
EC-2012^a^	MK392372 - MK392381	Ecuador	Al-Hussinee et al. (2018)
AD-2016^a^	KU552131 - KU552142	Israel	NCBI*
Til-4-2011^a^	KU751814 - KU751823	Israel	Bacharach et al. (2016a)
**TiLV/Israel/939-9/2018**	**OP037898 - OP037907**	**Israel**	**Current study**
**TiLV/Israel/939-16/2018**	**OP037908 - OP037917**	**Israel**	**Current study**
F3-4^a^	MK425010 - MK425019	Peru	Pulido et al. (2019)
TV1	KX631921 - KX631930	Thailand	Surachetpong et al. (2017)
TH-2013	MN687685 - MN687694	Thailand	Taengphu et al. (2020)
TH-2014	MN687695 - MN687704	Thailand	Thawornwattana et al. (2021)
TH-2015	MN687705 - MN687714	Thailand	Thawornwattana et al. (2021)
TH-2016-CN	MN687725 - MN687734	Thailand	Thawornwattana et al. (2021)
TH-2016-CU	MN687715 - MN687724	Thailand	Thawornwattana et al. (2021)
TH-2017	MN687735 - MN687744	Thailand	Thawornwattana et al. (2021)
TH-2018-K	MN687755 - MN687764	Thailand	Thawornwattana et al. (2021)
TH-2018-N	MN687745 - MN687754	Thailand	Thawornwattana et al. (2021)
TH-2019^a^	MN687765 - MN687774	Thailand	Thawornwattana et al. (2021)
WVL18053-01A^a^	MH319378 - MH319387	Thailand	Al-Hussinee et al. (2018)
WVL19031-01A^a^	MN193513 - MN193522	United States	Ahasan et al. (2020)
WVL19054^a^	MN193523 - MN193532	United States	Ahasan et al. (2020)

^a^ Reference TiLV complete genomes used for segment-specific primer set design.

*AD-2016 consists in 12 sequences where KU552135 and KU552139 are parts of ORF3 and KU552136 and KU552141 are parts of ORF5 and they were merged during the phylogenetic analyses.

**TABLE 2:** Designed primers for TiLV complete genome amplification.

Segment	Primer name	Primer sequence (5’ → 3′)	Amplicon size (bp)	Annealing temperature (°C)		Segment size (nt)
1	TiLV_1F_A	TTA​CGC​ACT​ATT​ACT​GTA​CTA​CCA	595	57		1,640
	TiLV_1R_A	AGA​TCT​AGC​GTG​CGT​CTC​TA				
	TiLV_1F_B	TGA​CGA​GCC​TGT​TGA​ACA​C	616	57		
	TiLV_1R_B	CGT​CGC​TGA​AAG​ACA​GGA​A				
	TiLV_1F_C	AAT​TGG​AGT​CAT​GCT​CGC​TT	730	57		
	TiLV_1R_C	TCC​AAG​TCT​GAG​AGA​GCC​TC				
	TiLV_1F_D	CAA​CCC​CAC​TTA​CAC​AAC​GA	440	57		
	TiLV_1R_D	GCA​AAT​ATT​TCT​CTC​ATT​CGC​CTA				
2	TiLV_2F_A	ACT​CTC​TAT​TAC​CAA​ATA​CAT​TTA​CT	664	56		1,470
	TiLV_2R_A	TTA​GCA​TCC​TCG​ACA​GCG​AC				
	TiLV_2F_B2	TCT​GGC​ACA​TGT​ATG​ACG​GG	587	57		
	TiLV_2R_B	AGG​CCC​TCT​ATC​GTA​ATG​TA				
	TiLV_2F_C	ATG​CAA​CAG​CTA​ACC​ACA​TA	633	55		
	TiLV_2R_C	TAC​CAT​ATA​TAT​AGT​GAA​GGC​TTT​TG				
3	TiLV_3F_A	CCC​CTT​AAT​CCT​TAA​TAG​ACC​G	566	55		1,370
	TiLV_3R_A	AGG​AAC​TTT​GAG​CAC​TCG​AA				
	TiLV_3F_B	GAC​GGG​GTT​GTT​AAA​GTT​GG	780	56		
	TiLV_3R_B	ATG​ACG​TCC​CAT​CTT​GTC​TC				
	TiLV_3F_C	GTT​GCT​TCT​CAT​AAG​CCT​GC	720	56		
	TiLV_3R_C	AAC​GTC​GTA​ACC​TTT​AGC​GA				
4	TiLV_4F_A	ACT​CCT​ATT​ACC​CAG​AAT​AGC​T	592	56		1,250
	TiLV_4R_A	CAA​ACT​GAC​GTA​CCT​AGC​CT				
	TiLV_4F_B2	GGA​TGA​GGG​TCG​GAA​GGA​GC	823	60		
	TiLV_4R_B2	CAG​CCT​GTG​CAG​CTT​TCC​G				
5	TiLV_5F_A2	ATC​TCA​GAC​TCC​AAT​AGC​TAT​GCA​G	703	60		1,130
	TiLV_5R_A2	CCG​GTG​ACT​TCC​CGT​GTC​AAA​G				
	TiLV_5F_B	GTG​GAC​GAC​TAC​AAG​ACC​AT	623	56		
	TiLV_5R_B	TGA​CCT​ACC​AGG​AAT​AGA​AGC				
6	TiLV_6F_A	CCA​AAT​TTT​ACC​TCT​CGC​ATG	714	56		1,040
	TiLV_6R_A	CTA​TTG​TCT​CTG​CAG​CTC​CA				
	TiLV_6F_B	GGA​TCA​AAA​GGG​GAA​CTC​CA	741	56		
	TiLV_6R_B	CAC​TTA​AAA​CTG​TAC​CTG​GGC				
7	TiLV_7F	TCT​CTT​TGC​ATT​GCA​TAC​CG	703	57		770
	TiLV_7R	AAC​TTA​GAA​AGG​CCT​CCC​CA				
8	TiLV_8F	TCC​AAT​TGG​ACA​GCA​TAT​CCA​GG	630	60		640
	TiLV_8R	AGC​TTA​CCT​CCC​TGG​GGA​AA				
9	TiLV_9F	TCC​GAT​TAC​TTT​TTC​CGC​TTG​G	453	58		548
	TiLV_9R	GGA​ATC​AGT​AGG​TTC​GCG​GA				
10	TiLV_10F	AAC​CCT​ACT​AAC​ACC​AAA​TAT​AGC​T	463	58		450
	TiLV_10R	TAG​TTA​GCG​TTG​GCC​TGT​GG				

### 2.5 Bioinformatic analyses

#### 2.5.1 Evaluation of the phylogenetic signal

Obtained sequences from isolates TiLV/Israel/939-9/2018 and TiLV/Israel/939-16/2018 were compared to 21 complete TiLV reference genomes (Table 1) currently available in GenBank (accessed on 01 May 2022). Sequences were aligned gene-by-gene using ClustalW method (Thompson et al., 1994) through MEGA 11 software (Tamura et al., 2021). The following analyses were carried out on both nucleotide and amino acid (aa) alignments. Nucleotide datasets were evaluated by single position (p001 = first position, p002 = second position, p003 = third position), combining first and second positions (p012), and by nucleotide-triplet (p123). Hence, five nucleic acids and one amino acids sequence alignments for each gene were tested. The analyses were performed to monitor the variability and the evolutionary rates among datasets in order to determine the best open reading frame ORF (or ORF combination) capable of elucidating the phylogenetic relationships between isolates. Firstly, from each dataset the p-distance and Maximum Composite Likelihood distances (Tamura et al., 2004) were pairwise estimated using MEGA 11 software. The evaluation of ratios between simple and complex distances showed average values close to 1 (>0.95), allowing to exclude the underestimation of variability in fast-evolving genes when considering the p-distances method (Nei and Kumar, 2000; Peer and Salemi, 2009). Concurrently, the likelihood mapping, performed using the Quartet Puzzling algorithm (Strimmer and Von Haeseler, 1997) available in IQ-TREE version 2.2 (Minh et al., 2020), allowed drawing all unique quartets and calculating the best evolutionary model for each ORF applying ModelFinder Plus (Kalyaanamoorthy et al., 2017). This strategy permitted to analyse the unrooted topologies inferred from all possible quartets of sequences in order to determine the phylogenetic signal among the datasets (Schmidt and von Haeseler, 2007; Strimmer and von Haeseler, 2009). Subsequently, the obtained average p-distances and the likelihood mapping were compared to detect the best candidate ORFs to perform phylogenetic analyses (Pereson et al., 2021) (Supplementary Figures S1, S2). Finally, we attempted to characterize the functional domains in TiLV ORFs using the Conserved Domain Database (CDD) (Marchler-Bauer et al., 2017; Lu et al., 2020) which includes data curated from both NCBI and additional resourses (Pfam, SMART, KOG, COG, PRK, and TIGRFAM). The analysis was performed using the amino acid sequence of each ORF as input for the CDD batch search tool available in NCBI database and applying a relaxed cut-off (E-value = 0.5). In order to avoid misidentification or false positives, results were considered acceptable only if more than 75% of the sequences indicated the same match (accession number and domain ranges). The same analyses were repeated by applying HMMER web tool (Finn et al., 2011; Potter et al., 2018) including the databases from UniProtKB, SwissProt, PDB and Ensemble.

#### 2.5.2 Phylogenetic analyses

The maximum likelihood (ML) phylogenetic approach involved multiple analyses on the best alignments (p123, see results section) and trees estimation was performed using IQ-TREE version 2.2. The best evolutionary models were determined as described above (Section 2.5.1) and were applied to run 25 independent analyses per ORF to minimise the possibility of being trapped in local optima. For each ORF of the 23 strains, the achieved topologies were compared *via* Robinson-Foulds distances (Robinson and Foulds, 1981), and trees with the best likelihood scores were set as starting points to evaluate the robustness. Statistical supports were determined for nodes and branches using 10,000 replicates in each analysis. While Bootstrap (BT) (Felsenstein, 2004) and the Ultrafast Bootstrap (UFB) (Hoang et al., 2018) analyses were applied to the nodes, the SH-like approximate likelihood ratio test (SH-alRT) (Guindon et al., 2010) was performed to the branches. The obtained topologies were also manually evaluated using Phylo.io application (http://phylo.io/index.html; accessed on 01 June 2022) which employs a colour scale highlighting the differences between compared trees (Robinson et al., 2016). Thus, the alignments of the best candidate ORFs were merged in progressive analyses by including or excluding ORFs through partition schemes (Chernomor et al., 2016) and discarding the ORFs which caused a decrease of support values. The combined phylogenetic analysis was carried out as described above and performed concatenating best candidate ORFs identified from previous findings. The phylogenetic tree was inferred from ORFs 1, 3 and 5 (p123) concatenated alignment (span respectively 1,557, 1,257, and 1,029 bps) applying the best evolutionary model (K2P + G4) as conveyed by the preliminary analyses and according to BIC (Bayesian information criterion) scores. The dataset was tested through 50 independent analyses and the edge-unlinked partition option (Lopez et al., 2002) was applied to compute the evolutionary models. Phylogenies were compared using the Robinson-Foulds distances and the tree with the best likelihood score was used to evaluate the robustness. Furthermore, a coalescent-based tree estimation was carried out to consider all the variability expressed by trees obtained from a single ORF (1–10). Hence, the gene-by-gene set unrooted tree previously described was processed using ASTRAL software with default settings and 10,000 replicates of BT (Mirarab et al., 2014). Nodes and branches supports (SH-alRT/UFB/BT) were considered significant when ≥ 90%. The phylogenetic tree was visualized with FigTree version 1.4.4 software (http://tree.bio.ed.ac.uk/software/figtree/; accessed on 01 May 2022) and was rooted in Peru (F3-4) strain which exhibited the highest average genetic distance.

To evaluate the different phylogenetic hypotheses represented by the trees of single ORFs, alternative topology tests were performed according to the approximately unbiased (AU) test (Shimodaira, 2002). Firstly, the test compared the topologies obtained from the single ORFs (1–10; Supplementary Figure S3) and the coalescent-based tree (Supplementary Figure S4B) with the concatenated dataset (ORFs 1, 3, and 5). Secondly, the same analysis was performed between the maximum likelihood tree (ORFs 1, 3, and 5; Supplementary Figure S4A) compared to the concatenated dataset of ORFs 1–10, in order to assure the method’s reliability, considering all available genetic variability among TiLV genomes. Finally, the maximum likelihood phylogenetic analysis was repeated, as described above, by removing two strains (TH-2018-K and TH-2016-CN) which increased the instability of the tree topology (see Section 3.5 Phylogenetic analyses).

#### 2.5.3 Reassortant detection analysis

For each strain of the dataset (total 23 strains), we produced a nucleotide sequence by concatenating the complete ORFs of each genetic segment. All concatenated sequences were then aligned as described above and checked for the presence of breakpoints events by applying five algorithms (RDP, GENECONV, Chimaera, MaxChi, and 3Seq) implemented in the Recombination Detection Program v.4.101 (RDP4) (Martin et al., 2015). Only potential reassortment events detected by more than three applied algorithms were considered acceptable (*p*-value ≤ 0.05).

## 3 Results

### 3.1 TiLV detection by rRT-PCR

Seven samples out of twelve, collected from the two Israeli farms, tested positive for TiLV by rRT-PCR targeting segment 3. Among the positive samples, only those showing the highest concentration of viral genetic material [in term of cycle threshold (Ct) values], and representing both farms, were selected for virus isolation prior to performing Sanger sequencing. Selected samples were TiLV/Israel/939-16/2018 (brain; Ct-value = 19.70) and TiLV/Israel/939-14/2018 (liver; Ct-value = 23.57) from farm D and sample TiLV/Israel/939-9/2018 (brain; Ct-value = 24.86) from farm J. All other positive samples gained Ct-values > 29 and consequently were excluded for subsequent analyses.

### 3.2 Virus isolation

**FIGURE 1:**
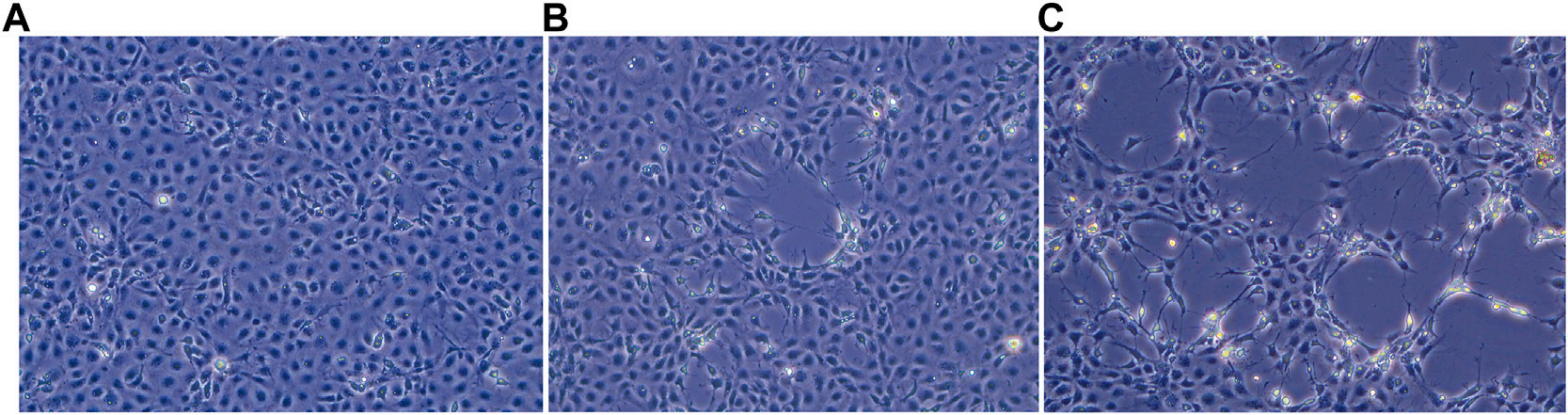
E-11 cell line observed under inverted microscope (Leica DMi1) equipped with camera (FLEXACAM C1, Leica) at ×20 magnification: **(A)** normal appearance of confluent monolayer at 48 h post seeding; **(B)** focus of cytopathic effect (CPE) at 48 h post infection; **(C)** extensive CPE after 72 h post infection.

The uninfected E-11 cell monolayer never showed any kind of cytopathic effect (Figure 1A). On the other hand, cytopathic effects were observed from all the samples 48 h post inoculum, and appeared as foci of infection with infected cell showing frayed cytoplasm and increased refraction (Figures 1B, C). Foci of infection rapidly merged at 72 and 96 h but never reached a complete detachment of the cell monolayer. The two isolates obtained from the brain samples (TiLV/Israel/939-9/2018 and TiLV/Israel/939-16/2018) were selected for subsequent sequencing and molecular characterization due to their faster growing behaviour and higher titres in comparison to the liver isolate.

### 3.3 TiLV genome segment sequencing

Individual genome, segments (1–10), of positive TiLV isolates obtained from the brain samples were amplified and Sanger sequenced. All assembled sequences are available from the GenBank database under the accession numbers OP037898 – OP037907 and OP037908 – OP037917 for isolates TiLV/Israel/939-9/2018 and TiLV/Israel/939-16/2018, respectively.

### 3.4 Evaluation of the phylogenetic signal

Results of average genetic variabilities obtained from all alignments (aa, p001, p002, p003, p012, p123) were compared into a scatter plot (Supplementary Figure S1). The graph was divided into three areas according to the different evolutionary rates. A first area featured a fast substitution rate and enclosed the average values of most ORFs obtained from the analysis of p003 alignment (p-distance ≥ 0.045). A second area, ranging between p-distance 0.025 and 0.045, mainly contained results regarding p123 alignment, except for ORFs 8, 9 and 10 which showed values just below the lower threshold, indicating a relative conservation degree of these ORFs. A third area (p-distance ≤ 0.025), characterized by a pronounced conservation level, hosted results derived from the remaining alignments (aa, p012, p001, p002) except for results from aa alignment of ORFs 5 and 6 (Supplementary Figure S1). Concurrently, the dataset was also analysed using the likelihood mapping approach to unveil the most informative markers (Supplementary Figure S2). Hence, alignments were evaluated to retrieve the proportion of fully resolved quartets (FRQ), partially resolved quartets (PRQ) and unresolved quartets (UQ). Overall, proportions were compared through histograms and results indicated that alignment p123 was often the subset with higher FRQ, similarly to ORF 6 (p002 and p003) alignments. Therefore, considering the p123 alignments, it was possible to split the results into three subgroups: i) ORFs with FRQ ≥ 75% (ORF 1, 3, 5, and 6); ii) ORFs with FRQ between 50% and 75% (ORF 2, 4, 7, and 9); and iii) ORFs with FRQ ≤ 50% (ORF 8 and 10) (Supplementary Figure S2). These findings suggested that the ORFs belonging to the first subgroup (FRQ ≥ 75%), although with UQ proportions around 20%, were suitable to achieve a stable relationship among isolates from a statistical point of view (Schmidt et al., 2002). Hence, ORFs 1, 3, and 5 were selected as the best candidates for the subsequent phylogenetic analysis, while ORF 6 was excluded as it displayed high values in terms of variability and FRQ. However, ORF 6 could be used to improve the relationship resolutions in sub-terminal or terminal level. Moreover, summary statistics related to single ORF’s (1–10) alignments regarding the 23 considered TiLV genomes were reported in Supplementary Table S1. Overall, these findings were also corroborated by the functional domain characterization of the ORFs composing TiLV genomes, although the results were mostly inconclusive. Indeed, considering the 23 sequences composing our dataset, only ORF 1 gained reliable results (E-value = 8.52E-10) and matched a RNA-dependent RNA polymerase, as previously reported by other authors (Bacharach et al., 2016a; Taengphu et al., 2020). However, isolate TH-2015 showed a significant shortening of the residues into the identified domain with a relative increase of the E-value. The other candidate ORFs achieved correlations to domains related with exonucleases activities (ORF 3: E-value = 0.43) and with the type II secretion system (ORF 5: E-value = 0.16). In case of ORF 6, only 19 isolates out of 23 displayed a correspondence with the immunoglobulin domain. In these last two ORFs (5 and 6), the domains detected by CDD analyses partially overlapped with residuals S5_196-272_ and S6_200-317_ which were demonstrated to have a substantial antigenic activity (Lueangyangyuen et al., 2022). No other domains were found for the remaining ORFs. Further results from the HMMER analyses did not provide any improvement, clearly recognizing only the domain already reported in ORF 1.

### 3.5 Phylogenetic analyses

The single-ORF phylogenetic analyses displayed uncertain and mostly unsupported topologies in the backbones and statistically supported UFB/BT values only in the terminal and sub-terminal nodes (Supplementary Figure S3). Furthermore, phylogenies of ORFs 8, 9, and 10 exhibited tree topologies with several polytomies, and hence supporting p-distance and likelihood mapping results that revealed high conservation levels of these ORFs. Similarly, Chaput et al. (2020) reported the same findings even though using a reduced dataset of only 9 isolates. The Israeli sequences under study (TiLV/Israel/939-9/2018 and TiLV/Israel/939-16/2018) clustered in a monophyletic group within a well-supported clade in trees of ORFs 1, 2, 4, 5, 6, 7, and 10. While considering the phylogenetic tree of ORF 3, they were demonstrated to be polyphyletic, suggesting the occurrence of a potential reassortment event (see Section 3.6 Reassortant detection analysis). As mentioned above, topologies gained from ORFs 8, 9, and 10 were discharged as they were affected by polytomies and unsupported nodes (Supplementary Figure S3).

**FIGURE 2:**
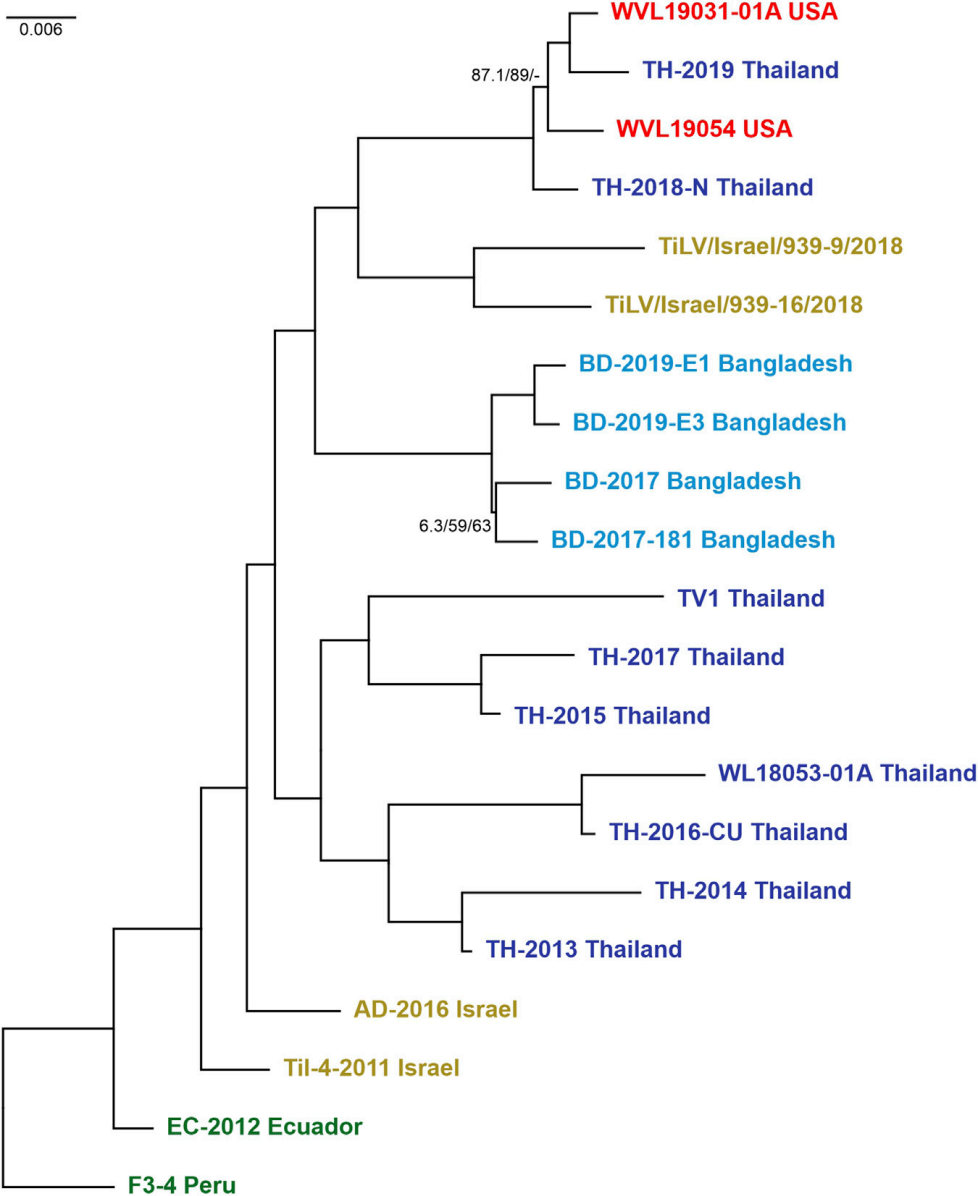
Maximum likelihood tree (-ln = 10614.8460) generated from the concatenated dataset of the 21 isolates (ORFs 1, 3, and 5). Values at the base of each clade correspond to SH-like approximate likelihood ratio test (SH-alRT)/Ultrafast Bootstrap (UFB)/Bootstrap (BT). Only values < 90 were reported showing the unsupported branches/nodes. Scale bar represents nucleotide substitutions per site. Isolates are colour coded according to belonging countries.

Preliminary phylogenetic analyses performed on the 23 strains and carried out on concatenated alignment of ORFs 1, 3, and 5 yielded a tree (here after fixed ML tree) topology with full support on most nodes and branches. However, while looking at the tree backbone, only two nodes exhibited lower support values and were associated with the split of Thailand isolates TH-2018-K and TH-2016-CN (MN687755/57/59 and MN687728/30/32) (Supplementary Figure S4A). Hence, the analysis was repeated by removing the latter mentioned strains from the concatenated alignment as probably involved in reassortment events. Actually, some reassortments were partially supported by evidences in both the single-ORF phylogenies and the RDP4 analyses. The final fixed ML tree topology of 21 strains (Figure 2) displayed a fully supported and stable structure, except for two terminal nodes showing lower supports. All the nodes in both trees were congruent, and the isolates clustered mainly into a monophyletic group based on “country of origin” rather than “year of isolation.” The only exceptions involved two Israeli isolates, Til-4-2011 (MK425010/12/14) and AD-2016 (KU552131/35/36) that showed a higher relation with the basal clade in comparison to recent sequences, as well as the hybrid cluster composed of isolates from Thailand and the United States, suggesting a probable translocation event of infected Tilapia between these two countries and thus corroborating the results from Ahasan et al. (2020). Moreover, the hybrid clade turned out to be a sister group of the clade containing isolates TiLV/Israel/939-9/2018 and TiLV/Israel/939-16/2018. Both these clades ended up forming a monophyletic group together with the clade of the Bangladeshi TiLV isolates, in which the sequences were split by years, respectively 2017 and 2019. Along the tree, a sister group of the already mentioned clades was found and contained the other isolates from Thailand. However, the latter monophyletic group appeared structured with two distinct lineages with an undetected common origin (Figure 2; Supplementary Figure S4A). The tree obtained by coalescence was mostly congruent with the fixed ML tree, although it exhibits lower BT values in the backbone (Supplementary Figures S4A, B). The relationships within and between clades were largely supported by findings shown in the coalescence-based tree, except for the main cluster of Thai isolates. Indeed, this group showed to be paraphyletic, with the clade containing TH-2014 Thailand and TH-2013 Thailand set basal and sister to all the other sequences from Thailand, Bangladesh, United States and the two new sequences from Israel. However, inference on the epidemiology of this viral species should be done with caution as obtained results are based on few sequences.

The test performed on alternative phylogenetic hypotheses demonstrated that i) the concatenated dataset (ORFs 1, 3, and 5) completely rejected the alternative topologies inferred from the single datasets (AU test, *p*-value < 0.05) while accepted the topology proposed by the coalescent-based tree (Supplementary Figure S4B); ii) conversely, the second analysis accepted the topology represented in the fixed ML tree (Supplementary Figure S4A) (AU test, *p*-value > 0.05), thereby describing the phylogenetic signal conveyed by the concatenated dataset of ORFs 1–10.

### 3.6 Reassortant detection analysis

**FIGURE 3:**
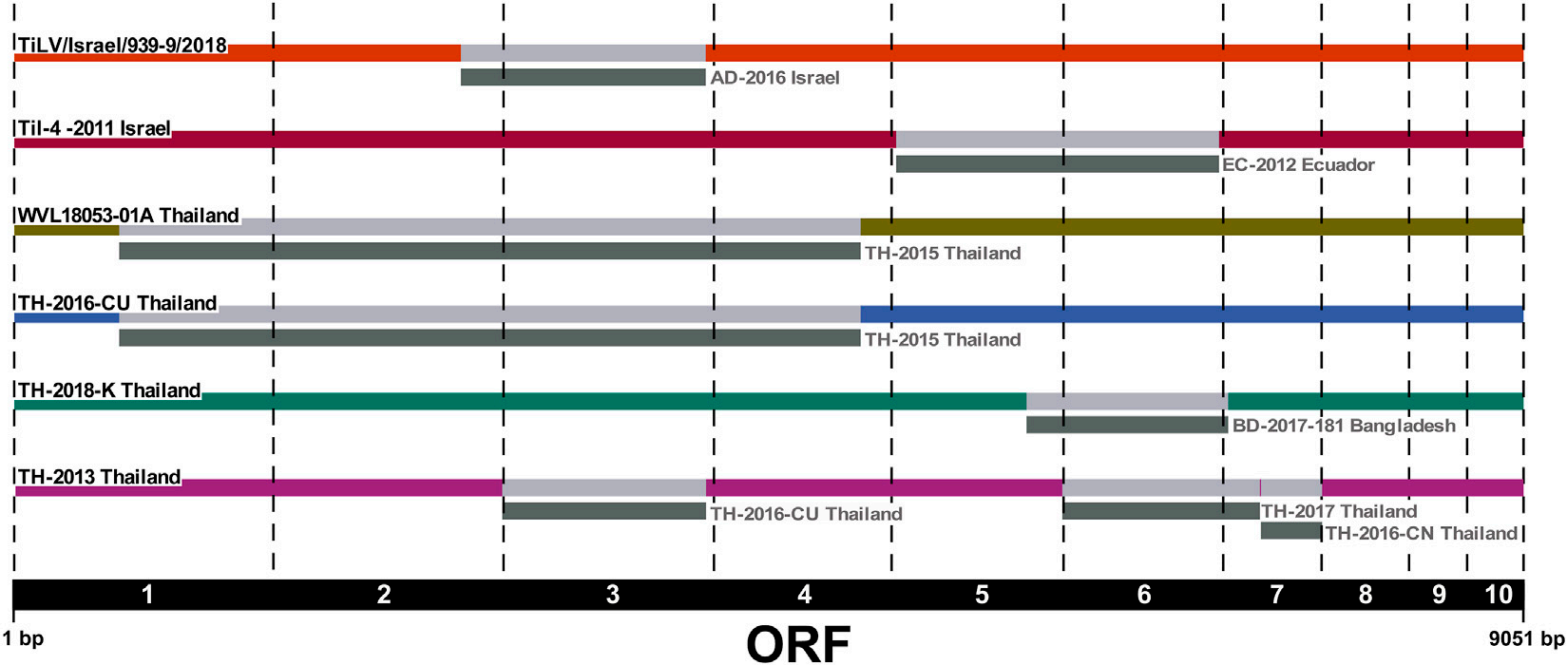
Reassortment breakpoint diagram of isolates detected by several methods implemented in RDP4 v.4.101 software. Reassortment events are coloured in grey. The ORF numbers (1–10) are reported below the diagram.

We applied the RDP4 software to the ORFs concatenated alignment in order to identify any evidence of reassortment among the newly sequenced Israeli TiLV segments and to confirm previously reported events. Our results highlighted the presence of two new potential reassortment events; the first one involving isolate WVL18053-01A which exchanged segments 1–4 with isolate TH-2015, and the second one involving the exchange of segment 3 between two Israeli isolates, namely, TiLV/Israel/939-9/2018 and AD-2016. Interestingly, in this latter case we reported for the first time a reassortment event of segment 3 in TiLV from Israel. Besides, our analysis found consistency with other potential reassortment events already described (Chaput et al., 2020; Thawornwattana et al., 2021; Verma et al., 2022). In fact, we identified the reassortment of segments 5 and 6 involving the Israeli isolate TiL-4-2011 and EC-2012, as well as the events identified in Thai isolate TH-2016-CU that involved exchanging segments from 1 to 4 with isolate TH-2015. Moreover, we reported the events involving isolate TH-2013 showing exchanging segment 3, 6, and 7 with isolates TH-2016-CU, TH-2017 and TH-2016-CN respectively. Lastly, a reassortment event of segment 6, involving isolate BD-2017-181, was identified in isolate TH-2018-K (Figure 3).

## 4 Discussion

TiLV is recognised as the causative agent of severe mortality events in both farmed and wild tilapia in many countries (Bacharach et al., 2016a; Kabuusu et al., 2018; Waiyamitra et al., 2021). Even though the virus has been discovered only in recent years, this pathogen has very likely been circulating in the aquatic environment for a long time (Jansen et al., 2019; Abdullah et al., 2022). Indeed, soon after the first report of TiLV in Israel in 2014 many tilapia-producing countries started communicating the presence and the circulation of the virus. Nowadays the presence of TiLV is generating concern and awareness worldwide, likely due to the rapid spread of the virus in a short time span and the dramatic consequences this could have on the global tilapia production (Aich et al., 2022). Interestingly and since 2017, the WOAH (former OIE) placed TiLV under observation and the virus has recently met all the criteria to become a listed disease. This means that TiLV will soon be included in “Chapter 1.3. Diseases listed by the WOAH—Listing of infection with Tilapia Lake Virus” (WOAH, 2022) underlining the importance of increasing and improving the knowledge on this viral pathogen.

Despite the economic importance of tilapia aquaculture, the scarce availability of TiLV complete sequences and epidemiological information are severely affecting the knowledge on the evolution, origin and widespread of this emerging pathogen. Moreover, the unknown function of almost all the genomic segments of this viral species makes it difficult to gain any consensus on the best fitting gene for assessing the relationship among isolates (Chaput et al., 2020). Actually, to date few works have attempted the genetic characterization and phylogenetic analysis of this virus, due to the limited complete sequences publicly available. Overall, these reports applied different approaches to determine the relationships among strains; nevertheless, they pointed out the importance of being cautious when performing phylogenetic analysis to track TiLV movements among countries, and—whenever possible—they recommended the use of whole genome sequences (Chaput et al., 2020; Thawornwattana et al., 2021; Verma et al., 2022). In our study, along with sequencing the complete genome of two Israeli viruses we also presented a multifactorial approach in order to perform a molecular characterization of each genetic segment by investigating the genetic differences (Supplementary Figure S1) and the phylogenetic signal present in each ORF (Supplementary Figure S2). Hence, obtained results allowed carrying out structured phylogenetic analyses with fixed and stable results (Figure 2; Supplementary Figure S4A). Such analyses provided information regarding the phylogenetic content and genetic distance of a sequence alignment considering the entire positions of single codons, single and combined positions within the same codon, as well as testing the signal carried by the amino acid sequences. Obtained data indicated the proportion of fully resolved quartets and highlighted higher percentages when considering the entire codon (p123), thus suggesting the suitability of these positions to infer further phylogenetic analyses (Supplementary Figures S1, S2). Overall, the achieved results allowed us to classify the ORFs into three groups: i) 1, 3, 5 and 6 (FRQ ≥ 75%), ii) 2, 4, 7 and 9 (50% < FRQ < 75%), and iii) 8 and 10 (FRQ ≤ 50%) (Supplementary Figure S2). Furthermore, the genetic variability analysis performed on each ORF made it possible to investigate their evolutionary rates and to discriminate them according to the substitution rates (Supplementary Figure S1). Finally, combining results from the likelihood mapping and p-distance analyses allowed the selection of candidate ORFs (1, 3 and 5) likely suitable to achieve a stable relationship among isolates (Supplementary Figures S1, S2, S4A). Our outcomes are in partial agreement with data previously reported by Thawornwattana et al. (2021), which describe how some segments (1, 2, 3, and 4) are able to confer much more power in order to achieve resolved phylogenies and robust evolutionary estimates.

Phylogenetic analyses on candidate ORFs were carried out considering both single and concatenated ORF alignments. Obtained trees from single ORF alignments were characterized by being unstable with some terminal or sub-terminal variations as well as low support values (Supplementary Figure S3) as it is often stated in previous works (Chaput et al., 2020; Thawornwattana et al., 2021). The best fixed ML tree topology with full supports on most nodes and branches was yielded when using the concatenated alignment of ORFs 1, 3, and 5 (Figure 2). In this tree topology, all isolates clustered into distinct monophyletic groups according to their country of origin. The only exception involved the cluster containing isolates from the United States (WVL19031-01A and WVL19054) and Thailand (TH-2018-N and TH-2019), and this event coincided with a recent report of a farm in the United States importing live tilapia from Thailand (Ahasan et al., 2020; Debnath et al., 2020). Furthermore, the isolate from Peru (F3-4) presented the highest genetic distance among strains, even if it was the most correlated to the isolates from Ecuador (EC-2012) and Israel (Til-4-2011 and AD-2016), and for this reason it was set as the root to the tree. Within the trees with the best topology, the two Israeli viruses under study clustered together as a sister group of the hybrid clade (United States and Thailand), far from the Israeli virus isolated in 2011 (Til-4-2011) (Figure 2; Supplementary Figure S4A), probably indicating that multiple TiLV introductions occurred in Israel over the last decade. This finding underlines once again the impact of new viral variants on fish trade in both virus free and already infected countries. The same results were obtained even when considering all available complete genomes (total 23 strains, Supplementary Figure S4A). Furthermore, the phylogenetic tree obtained using the entire dataset showed a correlation between TH-2018-K and the clade from Bangladesh, as well as TH-2016-CN and the cluster containing isolates from Israel, United States and Thailand. These results likely suggest a possible import of infected batches of fish from Thailand in both cases. However, due to the weak supports at the nodes related to these sequences and considering the variability of the topologies often due to partial dataset, further investigations and complete genomes are needed to confirm this finding, and generally, for a better comprehension of the evolution of the virus before inferring consideration from the phylogenetic trees.

Interestingly, the rejection of the alternative topologies confirmed the contrast between phylogenetic signals conveyed by a single ORF dataset, thus, supporting the application of a multigenic approach to perform phylogenetic studies on this viral species. Furthermore, the presented method produced a topology that fits the variability carried into the complete dataset (ORF 1–10) and firmly established the relationship between the isolates thus supporting its reliability.

Further, we also attempted to investigate the possible presence of genetic reassortment events both among the Israeli TiLV isolates under study as well as within the entire dataset. Actually, reassortment phenomena are widely common in viruses with a segmented genome, whereby strains exchange their genetic materials when co-infecting the same host cell (McDonald et al., 2016; Lowen, 2018). Several authors have recently identified the occurrence of genetic reassortment events also in TiLV strains from different regions, and suggested to take into account these phenomena while performing phylogenetic analysis (Chaput et al., 2020; Thawornwattana et al., 2021; Tran et al., 2022; Verma et al., 2022). Interestingly, in the present study we detected a new case of Israeli TiLV reassortant, namely, TiLV/Israel/939-9/2018, in which segment 3 was exchanged with another isolate from Israel (AD-2016). Moreover, our results were consistent with the findings reported in other studies (Chaput et al., 2020; Thawornwattana et al., 2021; Tran et al., 2022) regarding the exchange of segments 5 and 6 between the Israeli isolate Til-4-2011 and EC-2012 from Ecuador; in addition and according to Chaput et al. (2020), the history of these isolates appeared to include a relatively recent reassortment event. Herein, we also reported an exchange of segments from 1 to 4 between two Thai TiLVs (WVL18053-01A and TH-2015). Besides, we confirmed other potential reassortant TiLVs from Thailand that had already been identified in other studies. Indeed, we identified isolate TH-2016-CU in which reassortment involved exchanging segments from 1 to 4 with isolate TH-2015, as well as exchanging segments 3, 6, and 7 between isolate TH-2013 and TH-2016-CU, TH-2017 and TH-2016-CN, respectively. Finally, we also detected the exchange of segment 6 between isolate TH-2018-K and BD-2017 (Thawornwattana et al., 2021; Tran et al., 2022; Verma et al., 2022). Most likely, the reassortment events reported in TiLVs from Thailand, which led to the emerging of new variants, were caused by fish translocation activities between different regions of the country (Tran et al., 2022). Although the origins of such reassortants are still unclear, the driving force for such occurrences may be ascribable to the within-host reassortment due to the introduction of infected fish across regions throughout the intensive aquaculture practices (Verma et al., 2022). However, the identification of alternative reassortment events with respect to what reported in previously published studies is mainly due to the diversity of the dataset considered in our study. Indeed, the ability of the algorithms to identify breakpoint events depends on the presence, within an alignment, of highly diversified and of good quality sequences, as well as on the isolates representative of the ancestral sequences from which reassortants originate (Martin and Rybicki, 2000; Martin et al., 2015). Hence, more isolates from other geographical regions are required to determine the origin of the multiple reassortant genomes sampled over the years.

To conclude, in this study we presented a multifactorial approach aimed to determine the phylogenetic signal reported by each genetic segment in order to perform phylogenetic inferences on a set of selected candidate ORFs. Our results pointed out the suitability of using the concatenated ORF sets 1, 3, and 5, to perform maximum likelihood phylogenetic analysis yielding a fixed ML tree topology with full support on most nodes and branches. Finally, we again confirmed the ability of TiLV to reassort, a feature that further contributes to complicate the phylogenetic characterization of this virus. However, the insufficient number of publicly available TiLV complete genomes limits results reliability from any attempt of epidemiological surveillance aimed at increasing the knowledge about the prevalence, emergence and spread of the various TiLV strains. Hence, further investigations supported by a higher number of sequences from diverse regions are essential for the development of adequate surveillance strategies and for providing significant information on the epidemiology of this viral species.

## Data Availability

TiLV/Israel/939-9/2018 and TiLV/Israel/939-16/2018 complete genomes generated in this study can be found in online repositories. The names of the repositories and accession numbers can be found below: GenBank https://www.ncbi.nlm.nih.gov/genbank/, OP037898 – OP037907 and OP037908 – OP037917; European Virus Archive Global https://www.european-virus-archive.com/, Ref-SKU: 025V-04737 and 025V-04738.
