# Supplementary material for: Tilapia lake virus: A structured phylogenetic approach
Source: Front Genet. 2023 Apr 18;14:1069300. doi: 10.3389/fgene.2023.1069300 (PMC10151519; doi:10.3389/fgene.2023.1069300)
Supplement: Supplementary file 1 [file Table1.pdf]

Supplementary Table 1. Statistics for the single ORF (1 – 10) alignment regarding the 23 considered TiLV genomes

ORF	Alignment length (nt)	% Average alignemnt coverage	Average % GC content	% Informative sites	% Overall p-distances
1	1557	99,99%	48,9%	19,72%	3,70%
2	1371	99,96%	45,7%	19,26%	3,90%
3	1257	99,98%	48,0%	19,33%	3,90%
4	1062	100,00%	50,2%	15,91%	2,90%
5	1029	99,91%	46,4%	19,63%	3,70%
6	951	100,00%	43,5%	20,96%	4,60%
7	585	100,00%	49,7%	17,95%	3,60%
8	522	100,00%	46,7%	12,84%	1,80%
9	348	100,00%	48,1%	11,21%	1,80%
10	339	100,00%	47,4%	15,34%	2,40%
