# Supplementary material for: Tilapia lake virus: A structured phylogenetic approach
Source: Front Genet. 2023 Apr 18;14:1069300. doi: 10.3389/fgene.2023.1069300 (PMC10151519; doi:10.3389/fgene.2023.1069300)
Supplement: Supplementary file 2 [file Table1.DOCX]

| **ORF** | **Alignment length (nt)** | **% Average alignemnt coverage** | **Average % GC content** | **% Informative sites** | **% Overall**  **p-distances** |
| --- | --- | --- | --- | --- | --- |
| 1 | 1557 | 99,99% | 48,9% | 19,72% | 3,70% |
| 2 | 1371 | 99,96% | 45,7% | 19,26% | 3,90% |
| 3 | 1257 | 99,98% | 48,0% | 19,33% | 3,90% |
| 4 | 1062 | 100,00% | 50,2% | 15,91% | 2,90% |
| 5 | 1029 | 99,91% | 46,4% | 19,63% | 3,70% |
| 6 | 951 | 100,00% | 43,5% | 20,96% | 4,60% |
| 7 | 585 | 100,00% | 49,7% | 17,95% | 3,60% |
| 8 | 522 | 100,00% | 46,7% | 12,84% | 1,80% |
| 9 | 348 | 100,00% | 48,1% | 11,21% | 1,80% |
| 10 | 339 | 100,00% | 47,4% | 15,34% | 2,40% |

**Supplementary Table 1.** Statistics for the single ORF (1 – 10) alignment regarding the 23 considered TiLV genomes
