# Supplementary material for: Tilapia lake virus: A structured phylogenetic approach
Source: Front Genet. 2023 Apr 18;14:1069300. doi: 10.3389/fgene.2023.1069300 (PMC10151519; doi:10.3389/fgene.2023.1069300)
Supplement: Supplementary file 3 [file Image4.pdf]

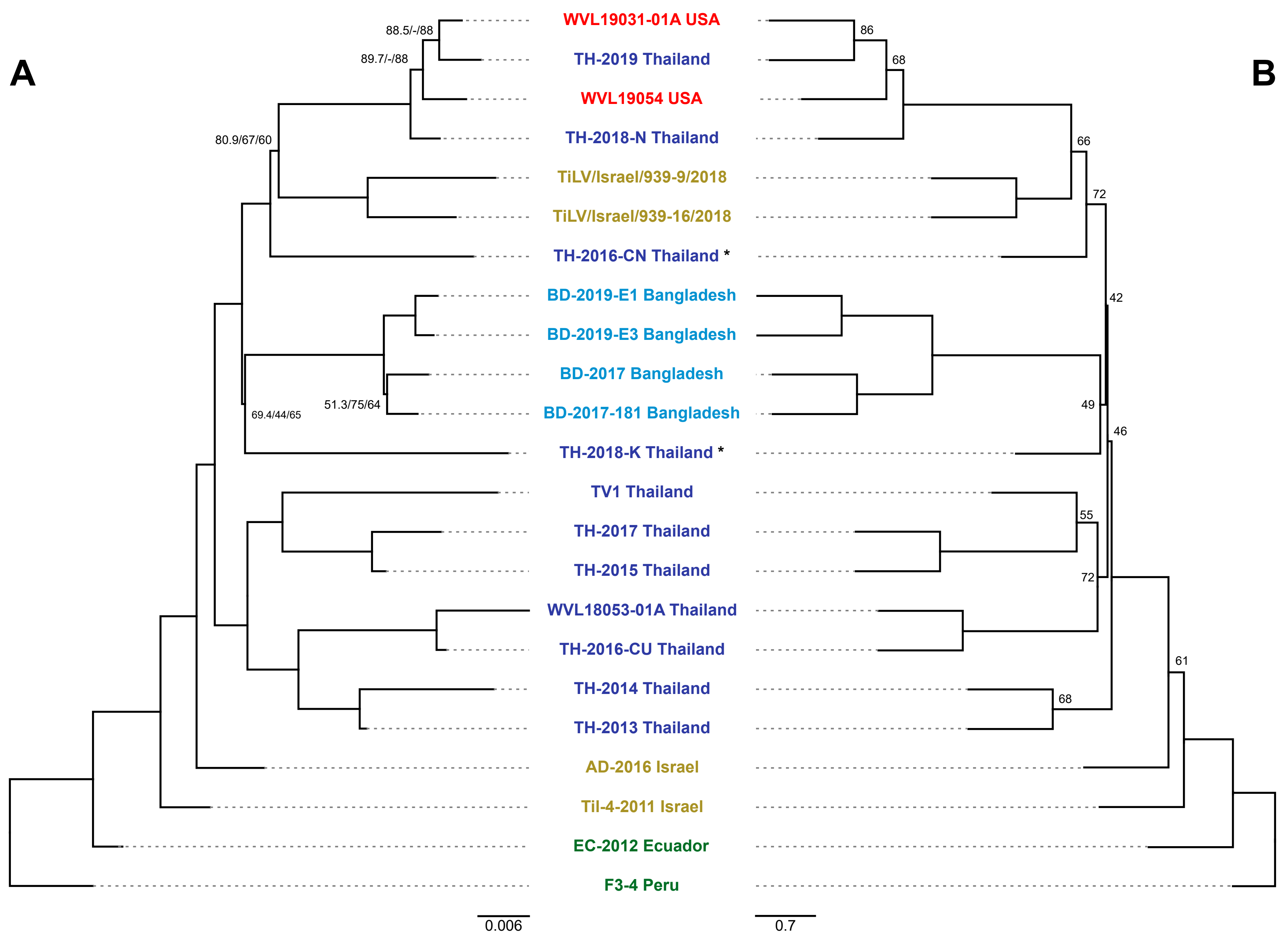

Figure S4. **A)** Maximum likelihood tree (-ln = 11487.8205) inferred from the concatenated dataset of the 23 isolates (ORFs 1, 3 and 5). Values at the base of clades correspond to SH-like approximate likelihood ratio test (SH-alRT)/Ultrafast Bootstrap (UFB)/Bootstrap (BT). **B)** Coalescent-based tree obtained using Astral software. Values at the base of clades correspond to the average Bootstrap (BT). For both trees, only values < 90 are reported showing the unsupported branches/nodes. *, indicates sequences removed from fixed ML tree (Figure 2). Scale bars represents nucleotide substitutions per site. Isolates are colour coded according to belonging countries.
