# Supplementary material for: Tilapia lake virus: A structured phylogenetic approach
Source: Front Genet. 2023 Apr 18;14:1069300. doi: 10.3389/fgene.2023.1069300 (PMC10151519; doi:10.3389/fgene.2023.1069300)
Supplement: Supplementary file 4 [file Image2.pdf]

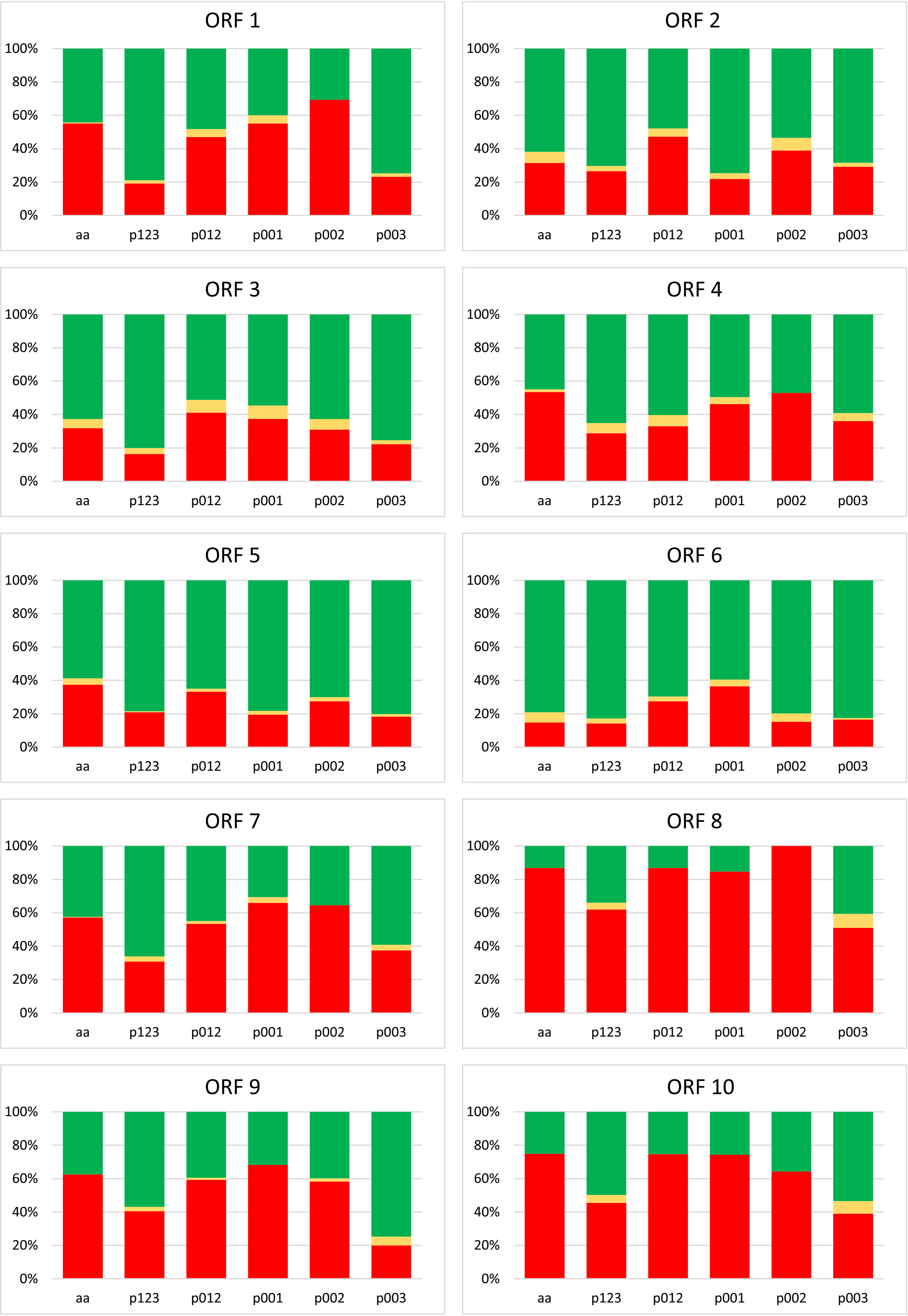

Figure S2. Likelihood mapping analyses carried out for amino acids (aa) and nucleotides sequences considering codons (p123) and single (p001, p002, p003) or combined nucleotide positions (p012) in the ORFs 1-10. Colour code indicates: ■, fully resolved quartets (FRQ); ■, partially resolved quartets (PRQ); ■, unresolved quartets (URQ).
