# Supplementary material for: Tilapia lake virus: A structured phylogenetic approach
Source: Front Genet. 2023 Apr 18;14:1069300. doi: 10.3389/fgene.2023.1069300 (PMC10151519; doi:10.3389/fgene.2023.1069300)
Supplement: Supplementary file 5 [file Image3.pdf]

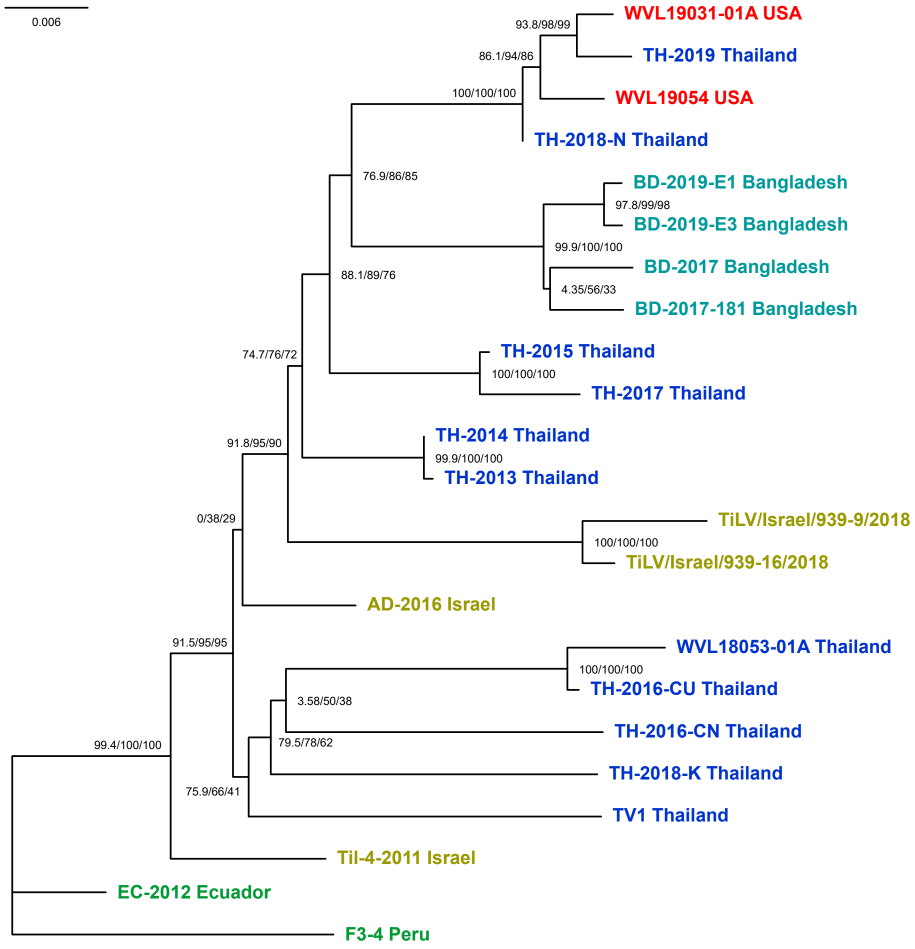

ORF1. Alignment length = 1557 bp. Best evolutionary model = K2P+G4. Log-likelihood score = -4573.4062.

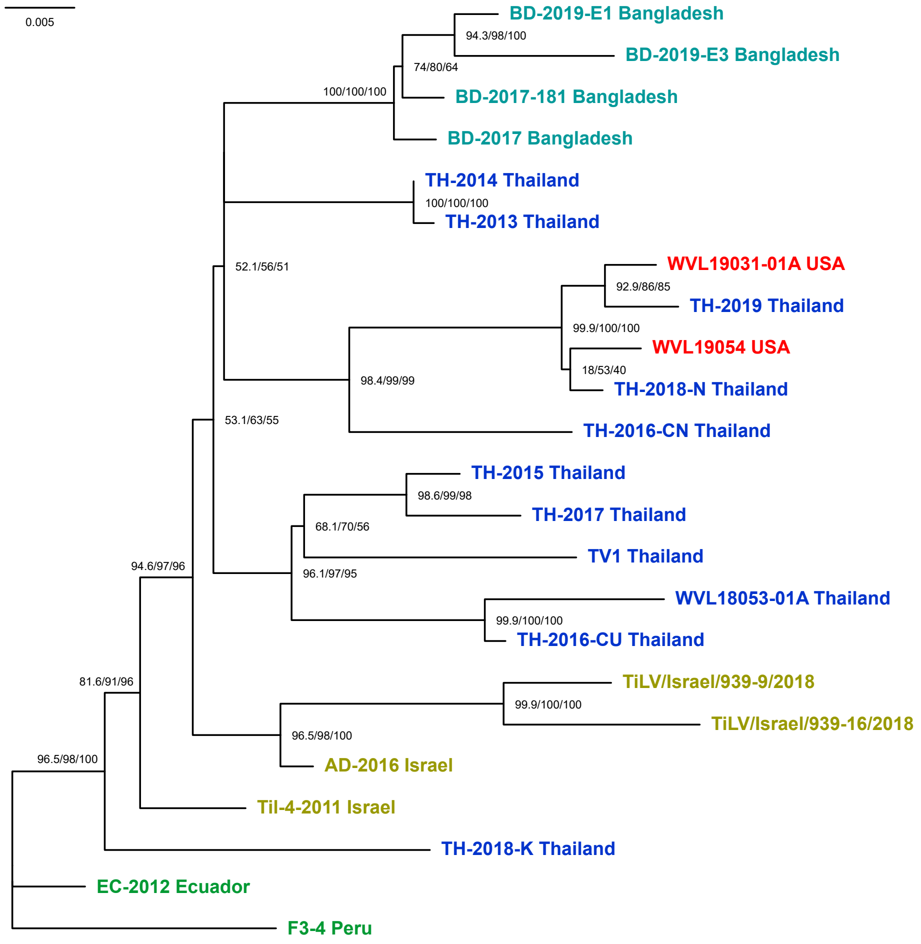

ORF2. Alignment length = 1371 bp. Best evolutionary model = K3P+G4. Log-likelihood score = -4118.6261.

Figure S3. Maximum likelihood phylogenetic trees inferred from single ORFs (1-10). Values on the splits indicate supports to the branches and to the nodes, respectively: SH-alRT / UFB / BT tests. Scale bar represents nucleotide substitutions per site. The caption under each figure reports information regarding the ORF alignment length, best applied evolutionary model and the obtained Log-likelihood score. Isolates are colour coded according to belonging countries.

(continue)

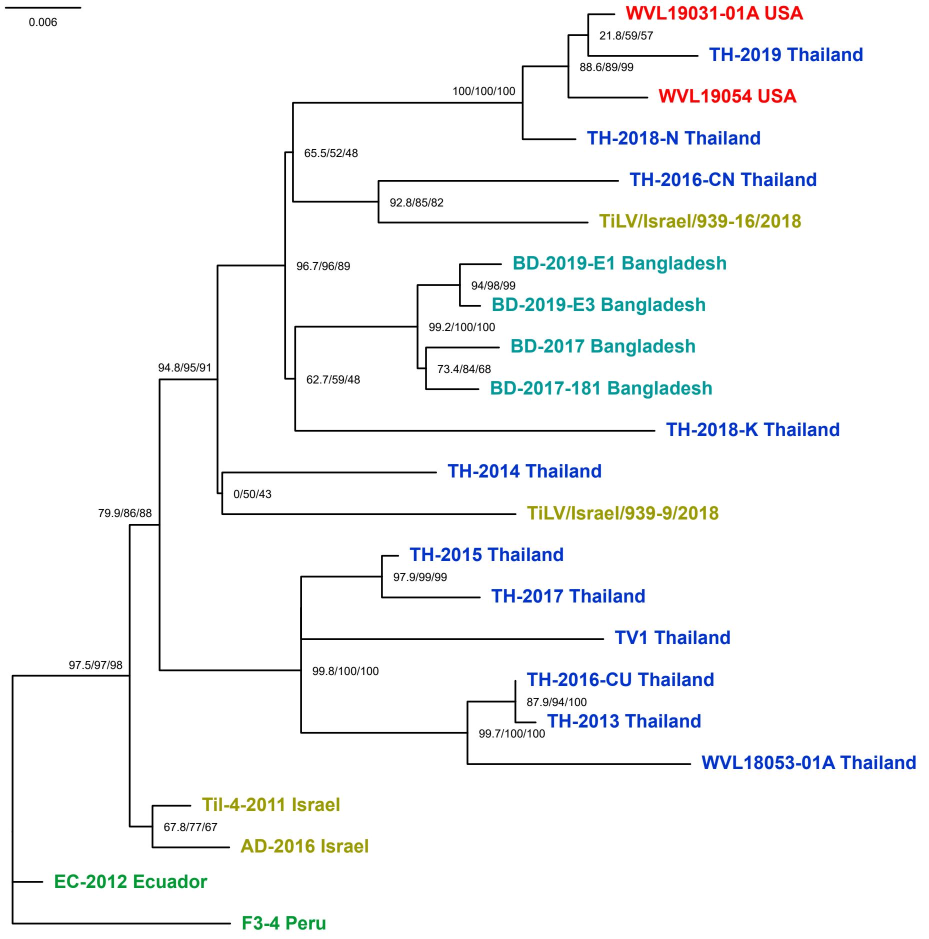

ORF3. Alignment length = 1257 bp. Best evolutionary model = K2P+G4. Log-likelihood score = -3800.6894.

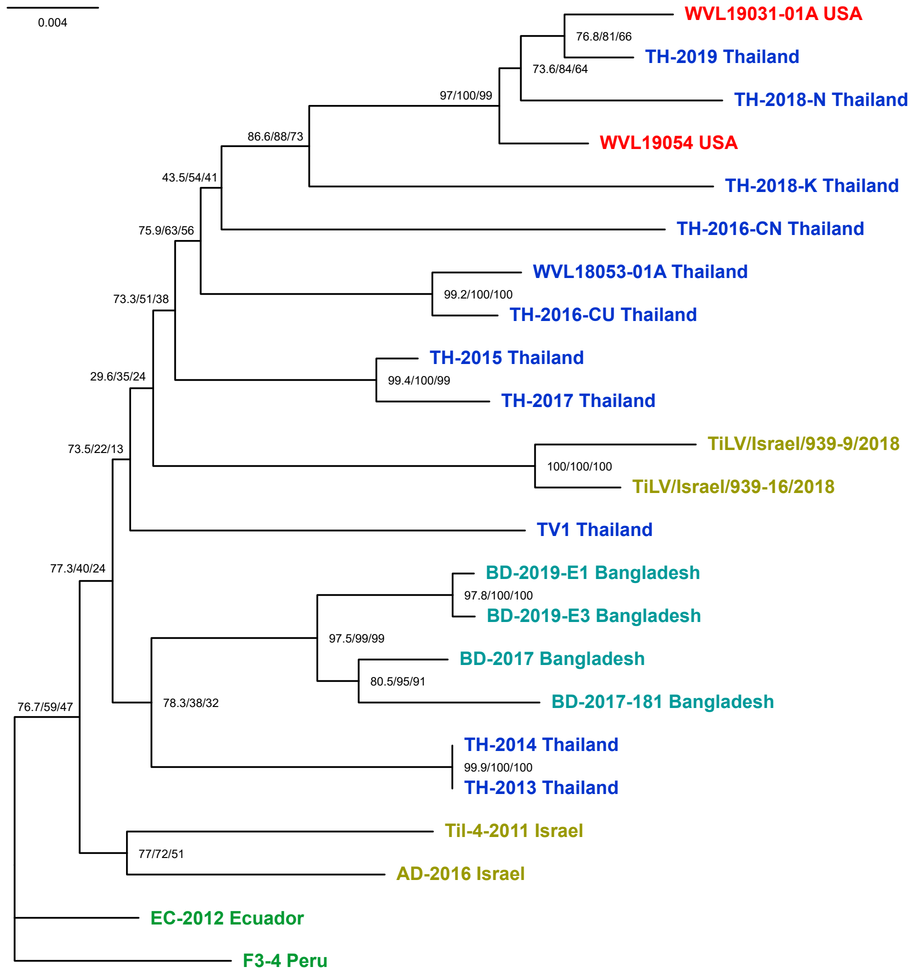

ORF4. Alignment length = 1062 bp. Best evolutionary model = K2P+G4. Log-likelihood score = -2887.5838.

Figure S3. Maximum likelihood phylogenetic trees inferred from single ORFs (1-10). Values on the splits indicate supports to the branches and to the nodes, respectively: SH-alRT / UFB / BT tests. Scale bar represents nucleotide substitutions per site. The caption under each figure reports information regarding the ORF alignment length, best applied evolutionary model and the obtained Log-likelihood score. Isolates are colour coded according to belonging countries.

(continue)

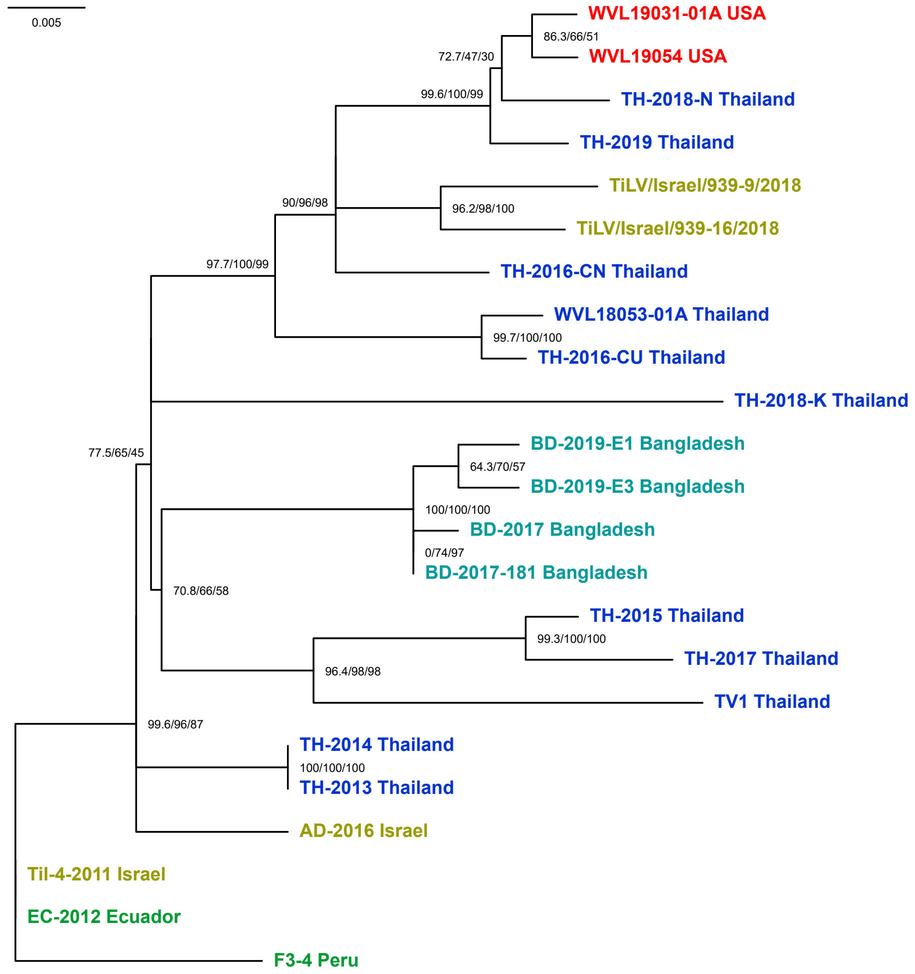

ORF5. Alignment length = 1029 bp. Best evolutionary model = K2P+G4. Log-likelihood score = -2978.7806.

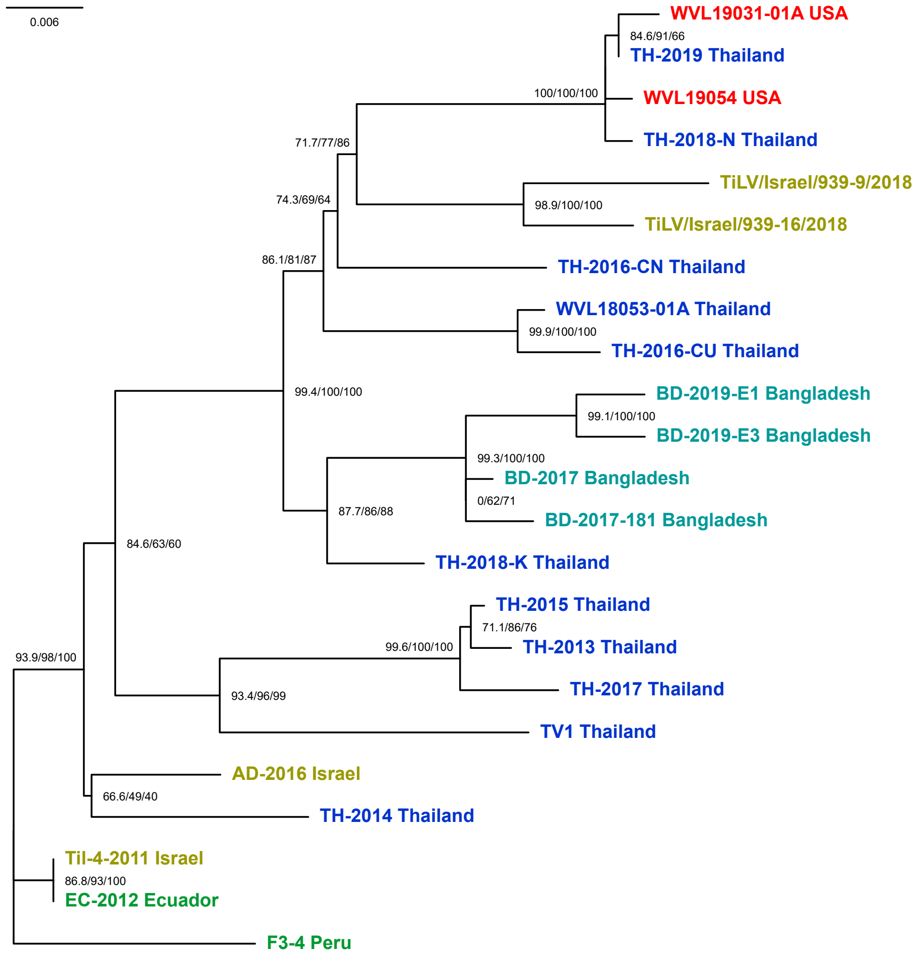

ORF6. Alignment length = 951bp. Best evolutionary model = HKY+F+I. Log-likelihood score = -2898.2547.

Figure S3. Maximum likelihood phylogenetic trees inferred from single ORFs (1-10). Values on the splits indicate supports to the branches and to the nodes, respectively: SH-alRT / UFB / BT tests. Scale bar represents nucleotide substitutions per site. The caption under each figure reports information regarding the ORF alignment length, best applied evolutionary model and the obtained Log-likelihood score. Isolates are colour coded according to belonging countries.

(continue)

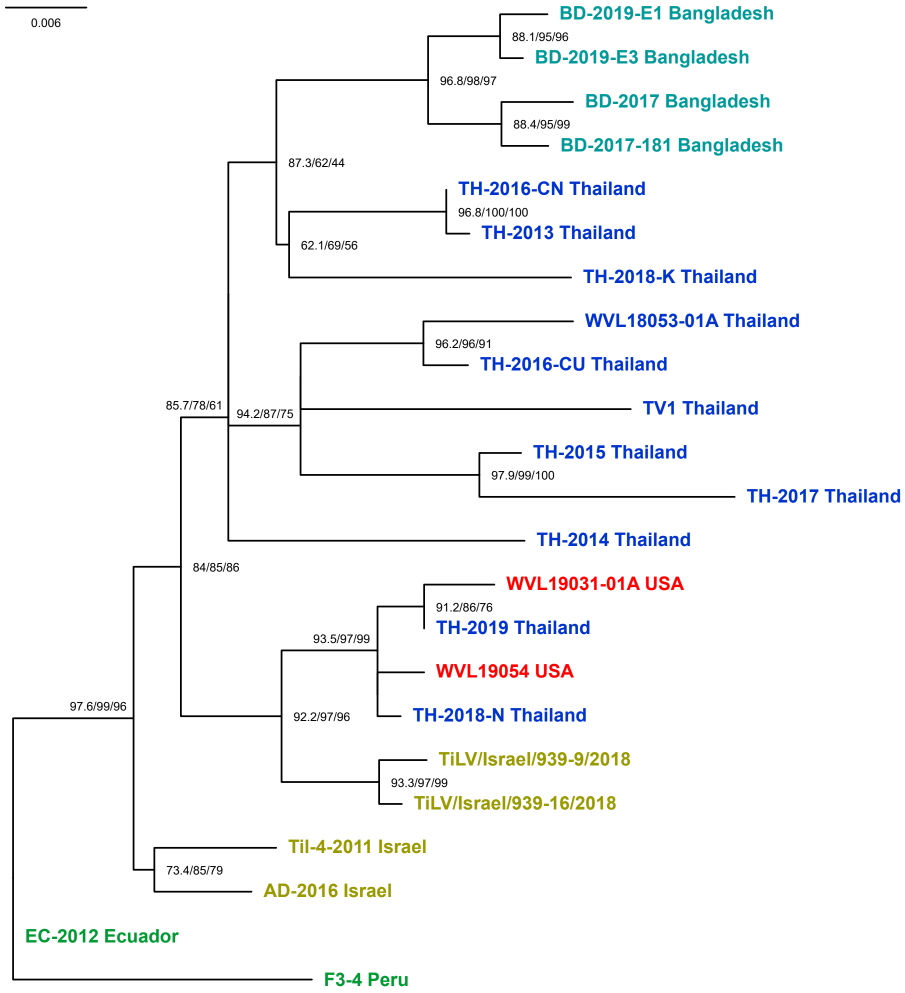

ORF7. Alignment length = 585 bp. Best evolutionary model = K2P+G4. Log-likelihood score = -1701.3697.

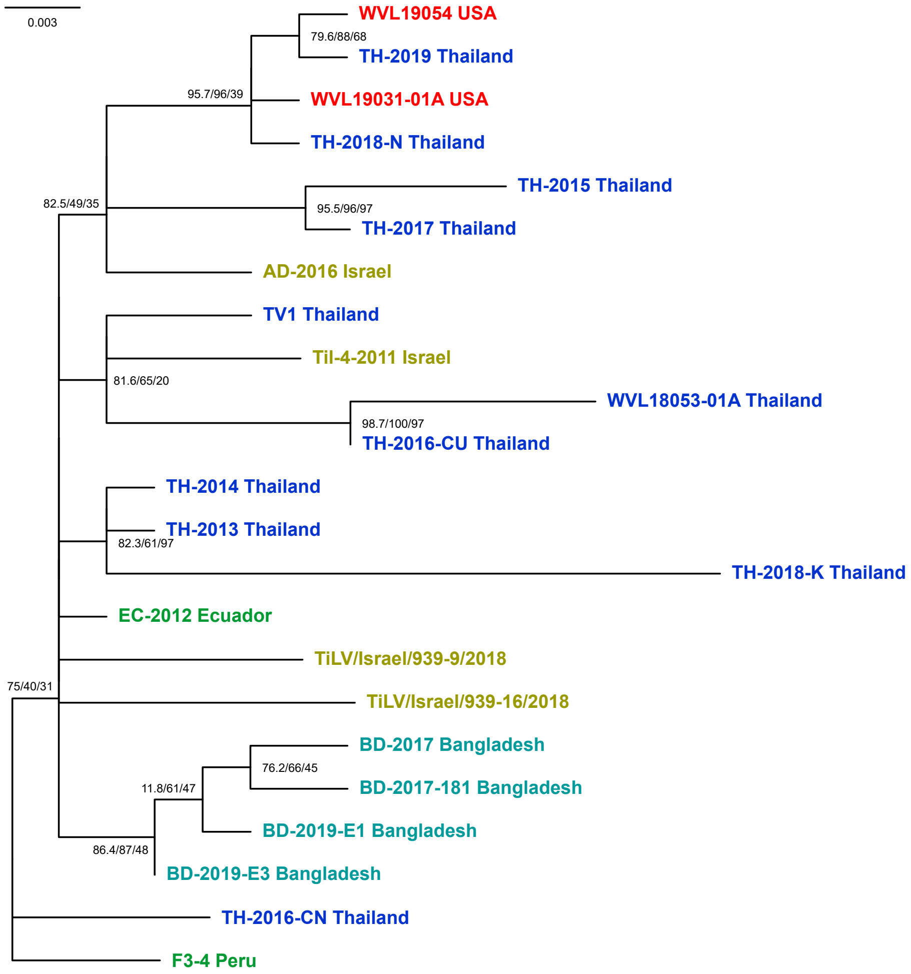

ORF8. Alignment length = 522 bp. Best evolutionary model = K2P+G4. Log-likelihood score = -1232.5907.

Figure S3. Maximum likelihood phylogenetic trees inferred from single ORFs (1-10). Values on the splits indicate supports to the branches and to the nodes, respectively: SH-alRT / UFB / BT tests. Scale bar represents nucleotide substitutions per site. The caption under each figure reports information regarding the ORF alignment length, best applied evolutionary model and the obtained Log-likelihood score. Isolates are colour coded according to belonging countries.

(continue)

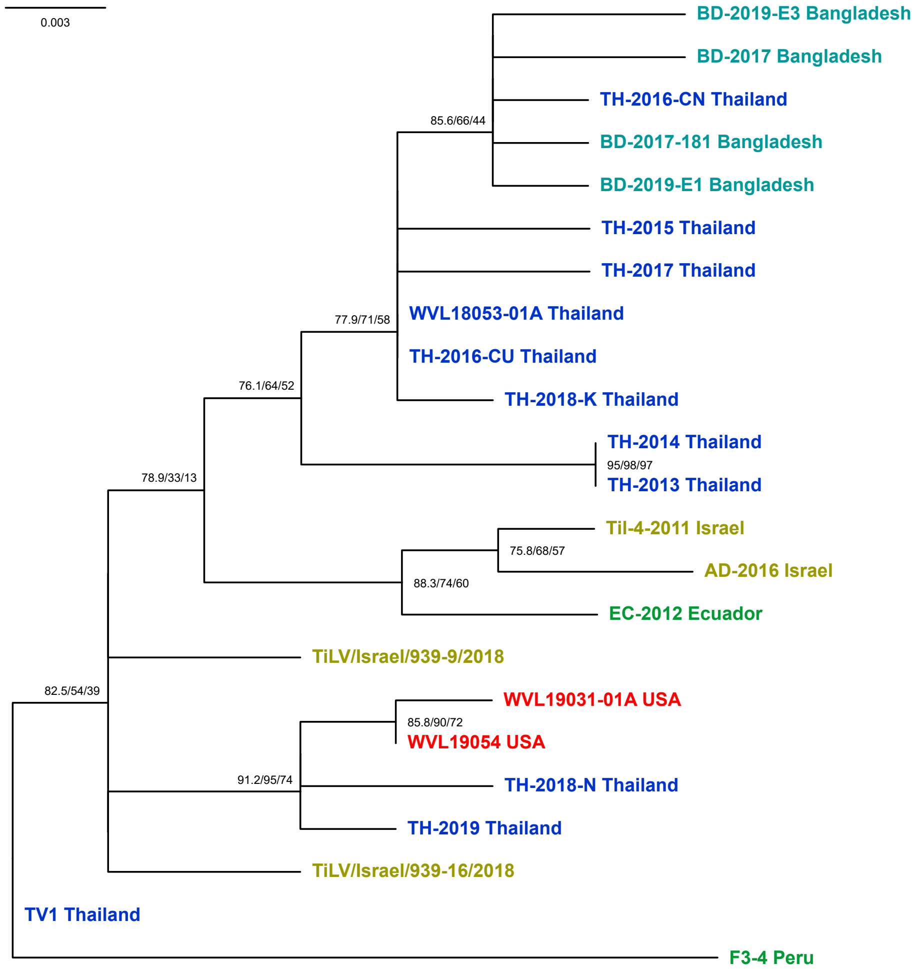

ORF9. Alignment length = 348 bp. Best evolutionary model = K2P+I. Log-likelihood score = -776.3058.

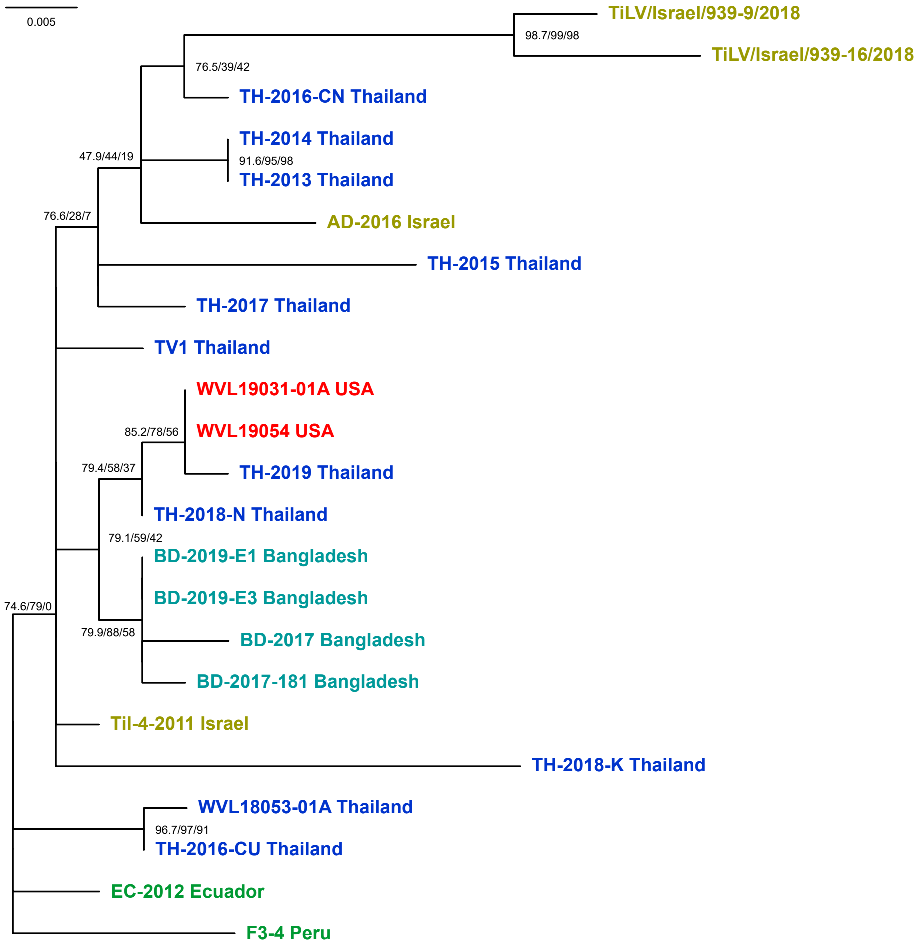

ORF10. Alignment length = 339 bp. Best evolutionary model = HKY+F+G4. Log-likelihood score = -841.3498.

Figure S3. Maximum likelihood phylogenetic trees inferred from single ORFs (1-10). Values on the splits indicate supports to the branches and to the nodes, respectively: SH-alRT / UFB / BT tests. Scale bar represents nucleotide substitutions per site. The caption under each figure reports information regarding the ORF alignment length, best applied evolutionary model and the obtained Log-likelihood score. Isolates are colour coded according to belonging countries.
