# Supplementary material for: Tilapia lake virus: A structured phylogenetic approach
Source: Front Genet. 2023 Apr 18;14:1069300. doi: 10.3389/fgene.2023.1069300 (PMC10151519; doi:10.3389/fgene.2023.1069300)
Supplement: Supplementary file 6 [file Image1.pdf]

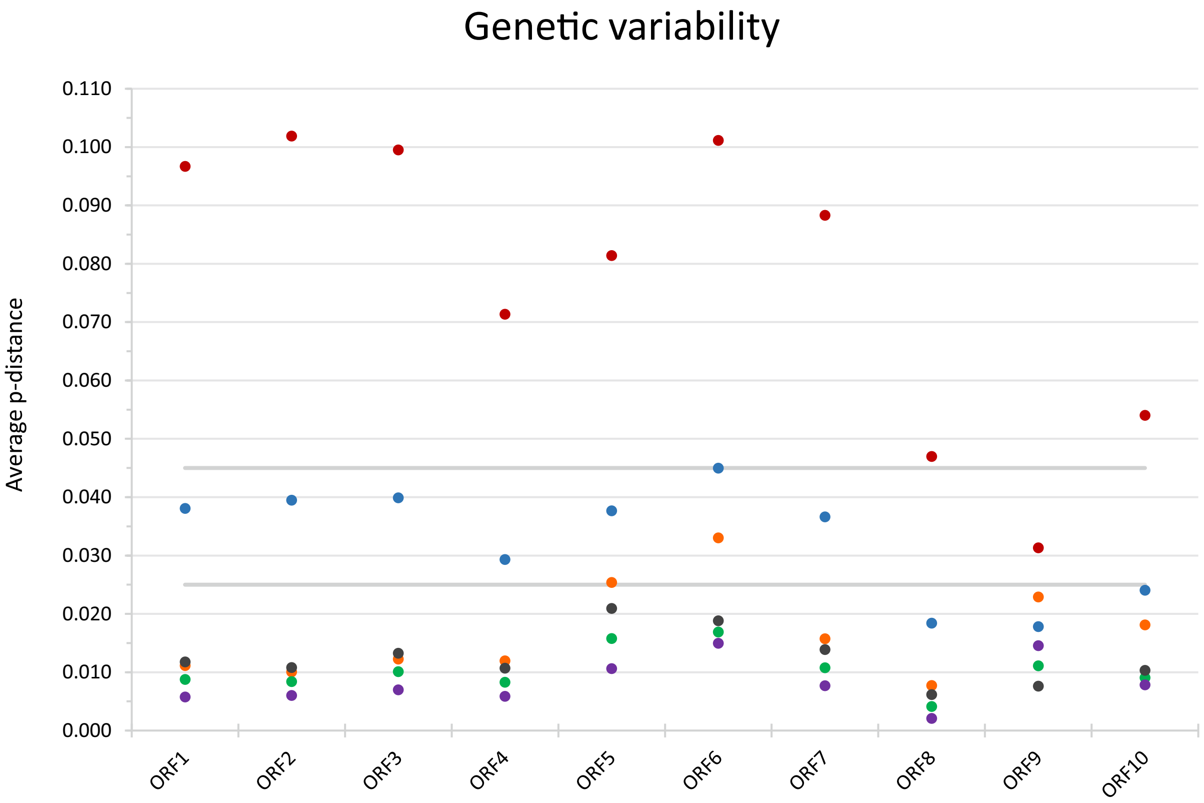

Figure S1: Genetic variability evaluation (p-distance) carried out for amino acids (aa, ●) and nucleotides sequences considering codons (p123, ●) and single (p001, ●; p002, ●; p003, ●) or combined nucleotide positions (p012, ●) in the ORFs 1 - 10.
